# The Renin-Angiotensin System: A Key Role in SARS-CoV-2-Induced COVID-19

**DOI:** 10.3390/molecules26226945

**Published:** 2021-11-17

**Authors:** George El-Arif, Antonella Farhat, Shaymaa Khazaal, Cédric Annweiler, Hervé Kovacic, Yingliang Wu, Zhijian Cao, Ziad Fajloun, Ziad Abi Khattar, Jean Marc Sabatier

**Affiliations:** 1 Department of Biology, Faculty of Sciences 2, Campus Fanar, Lebanese University, Jdeidet El-Matn 1202, Lebanon; george.elarif@gmail.com (G.E.-A.); antonella_farhat@hotmail.com (A.F.); 2 Department of Biology, Faculty of Sciences 3, Campus Michel Slayman Ras Maska, Lebanese University, Tripoli 1352, Lebanon; shaymaa.khazaal@hotmail.com; 3 Research Center on Autonomy and Longevity, Department of Geriatric Medicine and Memory Clinic, University Hospital, Laboratoire de Psychologie des Pays de la Loire, LPPL EA 4638, SFR Confluences, University of Angers, 44312 Angers, France; cedric.annweiler@chu-angers.fr; 4 Institute of NeuroPhysiopathology, Aix-Marseille University, CNRS, INP, 13385 Marseille, France; herve.kovacic@univ-amu.fr; 5 Modern Virology Research Center, State Key Laboratory of Virology, College of Life Sciences, Wuhan University, Wuhan 430072, China; ylwu@whu.edu.cn (Y.W.); zjcao@whu.edu.cn (Z.C.); 6 Azm Center for Research in Biotechnology and Its Applications, Laboratory of Applied Biotechnology (LBA3B), EDST, Lebanese University, Tripoli 1300, Lebanon; 7 Laboratory of Georesources, Geosciences and Environment (L2GE), Microbiology/Tox-Ecotoxicology Team, Faculty of Sciences 2, Lebanese University, Jdeidet El-Matn 1202, Lebanon

**Keywords:** SARS-CoV-2, COVID-19, ACE-2, Ang II/AT1R axis, RAS imbalance, cytokine storm, underlying diseases, genetic polymorphisms, host susceptibility factors

## Abstract

The novel severe acute respiratory syndrome coronavirus 2 (SARS-CoV-2), the causative agent of coronavirus disease 2019 (COVID-19), was first identified in Eastern Asia (Wuhan, China) in December 2019. The virus then spread to Europe and across all continents where it has led to higher mortality and morbidity, and was declared as a pandemic by the World Health Organization (WHO) in March 2020. Recently, different vaccines have been produced and seem to be more or less effective in protecting from COVID-19. The renin–angiotensin system (RAS), an essential enzymatic cascade involved in maintaining blood pressure and electrolyte balance, is involved in the pathogenicity of COVID-19, since the angiotensin-converting enzyme II (ACE2) acts as the cellular receptor for SARS-CoV-2 in many human tissues and organs. In fact, the viral entrance promotes a downregulation of ACE2 followed by RAS balance dysregulation and an overactivation of the angiotensin II (Ang II)–angiotensin II type I receptor (AT1R) axis, which is characterized by a strong vasoconstriction and the induction of the profibrotic, proapoptotic and proinflammatory signalizations in the lungs and other organs. This mechanism features a massive cytokine storm, hypercoagulation, an acute respiratory distress syndrome (ARDS) and subsequent multiple organ damage. While all individuals are vulnerable to SARS-CoV-2, the disease outcome and severity differ among people and countries and depend on a dual interaction between the virus and the affected host. Many studies have already pointed out the importance of host genetic polymorphisms (especially in the RAS) as well as other related factors such age, gender, lifestyle and habits and underlying pathologies or comorbidities (diabetes and cardiovascular diseases) that could render individuals at higher risk of infection and pathogenicity. In this review, we explore the correlation between all these risk factors as well as how and why they could account for severe post-COVID-19 complications.

## 1. Introduction

Coronaviruses are members of *Coronaviridae*, a heterogeneous family of enveloped RNA viruses causing respiratory and enteric diseases in humans. Three potentially fatal diseases in humans caused by novel coronaviruses have been identified so far: the severe acute respiratory syndrome (SARS) in 2002, the Middle East respiratory syndrome (MERS) in April 2012, and lately, the coronavirus disease 2019 (COVID-19), caused by SARS-CoV, MERS-CoV and SARS-CoV-2, respectively [1]. The novel coronavirus was shown to have similarity to its counterpart in bats, considered as the primary source of the virus, which could then have spread to humans either directly or from wild animals as intermediate hosts [2,3]. COVID-19 was first reported from Wuhan, China in December 2019, and spread worldwide two months later. Around 200 countries over the entire world have reported different numbers of cases. The disease has drastically expanded in the United States, Spain, Italy, Germany, France, China, Iran, the United Kingdom and Turkey [1]. 

An array of clinical manifestations that vary in severity, from asymptomatic and very mild (fever and respiratory symptoms such as cough and shortness of breath) to severe (pneumonia, acute respiratory disease syndrome, or ARDS, and total organ failure) are associated with COVID-19 [1]. Presently, over 250 million people worldwide have contracted COVID-19, and more than five million have died as a result of infection with SARS-CoV-2 [1]. Elderly persons and those suffering from co-morbidities such as heart disease, lung disease, diabetes, hypertension and coronary artery disease (CAD) are at higher risk of developing severe COVID-19 illness. On 18 March 2020, a COVID-19 Response Team reported that 80% of COVID-19-related deaths were among the elderly, aged >65 years [1,4]. The main cause of death in COVID-19 patients is inflammation, especially in the lungs, which leads to ARDS [5]. Unfortunately, no specific treatment is available for fatal and fast-spreading SARS-Cov-2 infection. However, a limited number of clinical studies have reported that some medications, such as antimalarial drugs (chloroquine and hydroxychloroquine), antiviral drugs (remdesivir) and dexamethasone (corticosteroid) have the potential to reduce the duration and symptoms of COVID-19 [6]. Indeed, patients treated with chloroquine (CQ) recovered much sooner than other patients cured with other medicines [7]. Furthermore, the treatment with hydroxychloroquine (HCQ) ameliorated the fever and decreased the cough duration [8]. However, treatment with HCQ and CQ combined was not effective. A recent study by Hadjadj et al., found that patients with severe COVID-19 had impaired type-I interferon (IFN-I) activity, increased T cell apoptosis and inflammatory response, while the regulatory effect on the immune response by HCQ/CQ was not powerful enough to inhibit the over-activation of the innate immune system [9]. Other findings suggested that SARS-CoV-2 can escape lysosome destruction by blocking the fusion between autophagosomes and lysosomes [10,11]. In addition, it has been reported that montelukast, a drug usually used in asthma, may be also proposed as an adjuvant in COVID-19 therapy due to its several beneficial properties (antiviral and antioxidant properties, prevention of neurological disorders due to SARS-CoV-2, limitation of the ischemia and the cytokine storm, improvement of respiratory symptoms) [12] as well as dexamethasone, which could provide a beneficial effect in COVID-19 patients [13]. 

SARS-CoV-2 single-stranded RNA genome encodes for the non-structural proteins (Nsps) and for four structural proteins: Nucleocapsid (N), Envelope (E), Membrane (M), and Spike (S), as well as the open-reading frame (ORF1a and ORF1ab) precursor polyproteins [14]. The Nsp1 is considered to be a major virulence factor which blocks the translation of the messenger RNA through binding to the small ribosomal subunit, thus inhibiting the production of host proteins, including interferons. Therefore, Nsp1 favors viral replication by destroying the innate immunity defense mechanisms [14]. However, the viral surface spike glycoprotein called “S-protein”, consisting of two domains (S1, S2) separated by a protease cleavage site [3], is considered the key element of viral–host interaction by facilitating the virus’ entry into the hot cells [3,15]. In fact, the viral entry is mediated by the binding of the S1 domain to the host cell surface receptor “angiotensin converting enzyme 2 (ACE2)”, a major component and a key player of the Renin–Angiotensin system (RAS) [15,16]. The importance of ACE2 in the pathogenesis of SARS-CoV-2 infection has been of particular interest in recent months. The successful binding of the S1 receptor-binding domain (RBD) to the ACE2 peptidase domain (PD) will expose the S1/S2 inter-domain which, after cleavage by the host Transmembrane Serine Protease 2 (TMPRSS2), allows fusion of the viral and the host cell membranes, a mechanism mediated by the S2 domain [3,16]. Thus, SARS-CoV-2/RAS interaction is a key step favoring viral pathogenesis and COVID-19.

**Figure 1:**
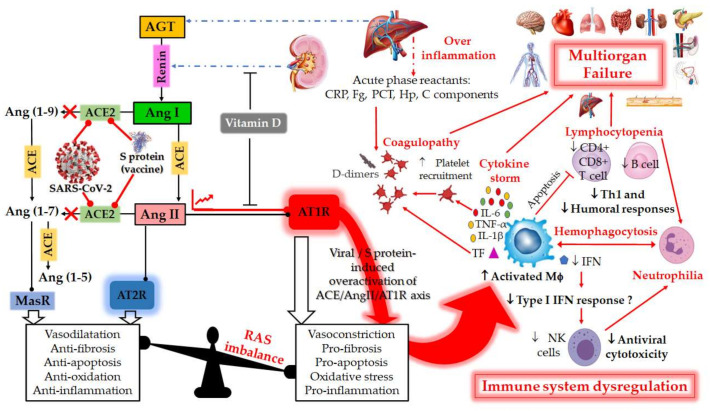
Schematic diagram of the dysregulation in the Renin–Angiotensin System (RAS) and the host immune system caused by the severe acute respiratory syndrome coronavirus 2 (SARS-CoV-2) or by vaccination with mRNA encoding SARS-CoV-2 spike (S) glycoprotein. RAS is a metabolic cascade which supports a series of enzymatic reactions in which the liver secreted AGT is transformed into Ang I by renin, which is a protease secreted by juxtaglomerular kidney cells in response to decrease in blood pressure or sodium load in the distal convoluted tubule. Ang I is subsequently converted to Ang II by ACE which can bind to the AT1R to exert several actions, such as vasoconstriction, pro-fibrosis, pro-apoptosis, oxidative stress and pro-inflammation. ACE2 counterbalances Ang II/AT1R effects by cleaving Ang I and Ang II into Ang-(1–9) and Ang-(1–7), respectively. Ang-(1–9) is also converted into Ang-(1–7), a negative regulator of the RAS, which binds to the MAS receptor to exert protective actions of vasodilatation, anti-fibrosis, anti-apoptosis, anti-oxidative and anti-inflammation. Ang-II can also bind to AT2R to counteract the aforementioned effects mediated by AT1R. The balance between the Ang II/AT1R axis and the ACE2/Ang (1–7)/MasR axis is therefore maintained under physiological conditions. However, during SARS-CoV-2 infection or upon receiving a spike protein-based vaccine, the viral Spike (S) glycoprotein binding to ACE2 receptor induces overactivation of the ACE/Ang II/AT1R axis. This event prevents normal Ang II degradation, the excess of which leads to AT1R overactivation and RAS system imbalance. Such an imbalance is very deleterious for the human body, mainly due to the important immunomodulatory roles of ACE2, which can directly interact with macrophages in the setting of vascular and lung inflammation. Patients with severe COVID-19 infections show hallmarks of sepsis, widely explained by an exacerbation of macrophage activation, including excessive inflammation with the presence of acute phase reactants (such as D-dimer, CRP, etc.), impending cytokine storms and overexpression of IL-1β, IL-2, IL-6, and TNF-α in the early phase of the disease. These induce the production of a compelling number of factors linked to the coagulation cascade (TF, Fb, etc.) and resulting in the onset of thrombi and associated disseminated intravascular coagulation (DIC). The inflammatory response to SARS-CoV-2 also consists of lymphopenia occurring early in >80% of patients and is prognostic, manifested as reduction in—and functional exhaustion of—CD4+ more than CD8+ T cells. Such impaired T cell responses can result from deficient IFN production, as IFNs act on the antigen-presenting cells, T cells, and induce other cytokines and chemokines that regulate T-cell responses. These events lead to imbalance of the innate/acquired immune response, delayed viral clearance and unusual predominance of hyperstimulated macrophage and neutrophil in targeted injured tissues. The permanent immune activation in predisposed elderly adults and patients with cardiovascular risk can lead to hemophagocytosis-like syndrome, with uncontrolled amplification of cytokine production, leading to endothelial dysfunction, tissue damage and multiorgan failure, which is the starting point of a progression towards the serious and fatal complications of COVID-19. This syndrome results from the ineffective activation of cytotoxic CD8+ T lymphocytes and Natural Killer T lymphocytes, and leads to ineffective viral cytotoxicity and weak antibody production. NK cells are regulated by IFNs during coronavirus infection, and patients with severe COVID-19 showed profound depletion and functional exhaustion of NK cells, the dysfunction of which could be due to dysregulation of IFN responses. On the other side, Vitamin D could help avoid the potential deleterious COVID-19 effects sometimes observed following vaccination, by either inhibiting renin secretion or suppressing AT1R overactivation. AGT: angiotensinogen; Ang I: angiotensin I; ACE: angiotensin-converting enzyme; Ang II: angiotensin II; ACE-2: angiotensin-converting enzyme-2; Ang 1–7: angiotensin 1–7; AT1R: angiotensin II type 1 receptor; SARS-CoV-2: severe acute respiratory syndrome coronavirus 2; COVID-19: coronavirus disease 2019; CRP, c-reactive protein; IL-1β, interleukin-1β; IL-6, interleukin-6; TNF-α. Tumor Necrosis Factor-alpha; INF, Interferon; Fg, fibrinogen; PCT, procalcitonin; Hp, haptoglobin; C, complement; M_Φ_, macrophage; NK, Natural Killer; Th1, T helper type 1; TF, tissue factor.

The RAS is a hormonal and physiological system whose function is to control blood pressure and circulating blood volume [17]. The ubiquitous RAS, also referred to as the Renin–Angiotensin–Aldosterone system, thus plays an essential function in humans as being a regulator of pulmonary, cardiovascular, renal and innate immune functions and the gut microbiota [18,19]. In the RAS pathway, renin secreted by the kidney cleaves Angiotensinogen (AGT) secreted by the liver to give Angiotensin I (Ang I). The latter is cleaved by the angiotensin-converting enzyme (ACE) to produce Angiotensin II (Ang II), which is the substrate of ACE2 (Figure 1). Ang II binds to the vasoconstriction-mediating type 1 (AT1R) and vasodilation-mediating type 2 (AT2R) receptors. Ang II can also be further cleaved by ACE2, into Angiotensin (1–7) [Ang-(1–7)], which interacts with the proto-oncogene G-protein-coupled receptor Mas (MasR) [19,20].

Recently, the dysregulation of RAS has been extensively investigated as being responsible for COVID-19 complex symptoms [17]. During viral infection, SARS-CoV-2 over-activates the RAS by binding to the ACE2 receptor, which normally has the function of degrading Ang II [21]. Thus, the binding of SARS-CoV-2 to the ACE2 receptor prevents the normal degradation of Ang II, the excess of which leads to over-activation of its cellular target, the AT1R. The over-activated AT1R is very deleterious for the human body, leading, in particular, to the appearance of COVID-19. The activated receptor induces vasoconstriction, hypertension, organ hypertrophy (heart, blood vessels), tissue fibrosis (heart, lungs, kidneys, and liver), ageusia (loss of taste), anosmia (loss of smell), neurological disorders, intestinal and vascular inflammation, obesity and action on glucose metabolism (diabetes), skin and testicular lesions [12,17,20,22] (Figure 1). The location of ACE2 within the body is thought to be key to determining the progression of disease by viruses targeting this receptor, given the fact that SARS-CoV-2 relies on the binding of the viral S glycoprotein to ACE2 for viral entry into cells. Consequently, ACE2 would also be downregulated by SARS-CoV-2 in COVID-19 [23]. Since the RAS has a central role in COVID-19 diseases, the selective targeting of its major components could be an essential treatment measure [19]. Recently, there are suggestions to use AT1R antagonists as a strategy of increasing ACE2 levels for treating SARS-CoV-2 infections [24,25]. Treatments with AT1R antagonists and vitamin D supplementation ensure a simpler and effective way of improving ACE2 levels independently among COVID-19 patients. Moreover, Vitamin D has a negative regulatory role on the RAS [19]. Suppression of RAS by AT1R antagonists results in a feedback-induced compensatory increase in renin. Vitamin D suppresses the compensatory increase in renin levels following the inhibition of the RAS by AT1R antagonists [26].

In this review, we will highlight the relationship between COVID-19 and RAS. The correlation between AT1R function and its over-activation by the downregulation of ACE2, which is blocked by SARS-CoV-2 infection, will be discussed. Particularly, we will focus on whether the RAS polymorphism could be associated with the different symptoms of COVID-19 as well as on the effects of host related factors on the virus clinical outcome.

## 2. RAS and COVID-19

### 2.1. RAS: A Portal Entry for SARS-CoV-2

The RAS is a metabolic cascade involved in the regulation of cardiovascular homoeostasis and cardiac remodeling [27]. Besides regulating blood pressure and circulating blood volume as well as fluid and salt balance [28,29], this system also has a significant role in atherosclerosis pathogenesis and in endothelial dysfunction [17,30]. RAS major components are renin, angiotensin, AGT, ACE and AT1R [31]. A homologue of ACE, named ACE2, with opposite effect, has been identified and considered to be a key player in the RAS [32,33]. ACE2 protein, a transmembrane metallo-carboxypeptidase of type I, is mainly found on the renal epithelium, endothelial cells of vasculature and Leydig cells of testes [34]. Thus, ACE2 expression is ubiquitous, being also included in the lungs, gastrointestinal (GI) tract [34] and the central nervous system, where it is involved in central regulation of the cardiovascular function [27]. ACE2 is implicated in regulating blood pressure and the hemostasis of electrolytes and fluid, with functional influences on different organs such as blood vessels, heart, kidneys and eyes [35]. Pathologically, ACE2 has also been associated with hypertension, stroke, dyslipidemia, cardiovascular and kidney diseases [34].

The balance of ACE and ACE2 regulates the physiological homeostasis of this system. In fact, RAS supports a series of complex enzymatic reaction in which ACE transforms Ang I to a vasoactive vital peptide Ang II [32,33,36]. Two types of receptor for Ang II have been identified: AT1R and AT2R [37,38]. Ang II, considered as a strong vasoconstrictor peptide which applies its function through the AT1R [38], favors a strong vasoconstriction and induces the profibrotic, proapoptotic and the proinflammatory signalizations in lungs and other organs [36]. In addition, Ang II–AT1R binding activates many cascades in the vasculature, such as protein tyrosine phosphatases, nicotinamide adenine dinucleotide phosphate (NADPH) oxidase, mitogen-activated protein (MAP) kinases and NO synthase, leading to the expression of proinflammatory mediators, contraction and endothelial dysfunction [30]. However, ACE2 counterbalances Ang II–AT1R effects by either stimulating an alternative pathway for Ang I degradation to produce Ang-(1–9), or by inactivating Ang II and hydrolyzing it to a heptapeptide Ang-(1–7). The latter stimulates vasodilation and activates anti-inflammatory, anti-fibrotic and anti-thrombotic cascades via the MasR axis [32,36], as well as the protection of endothelial cell activity [30]. Indeed, it has been shown that in failing hearts, ACE2 level was upregulated, and that ACE2-lacking mice showed damage to heart contractility and renal diseases, implicating an essential role of ACE2 in the regulation of Ang II biological activity [32]. Therefore, ACE2 loss may minimize the degradation of Ang II and lead to vasoconstriction and oxidative stress [33].

Interestingly, RAS has been shown to be involved in the pathogenesis of COVID-19, since the causative SARS-CoV-2 virus employs the human ACE2 cell surface protein as a receptor to invade host cells [3,34,35,36]. The first step of a viral infection is the entry into host cells, realized by the binding of a spike glycoprotein on the viral envelope to ACE2 receptors on host cell membrane [39]. Several studies have shown that SARS-CoV-2 can penetrate cells through ACE2 expressing cells, but not cells without ACE2 [40]. Also, binding kinetics revealed a 10 to 20-fold higher binding affinity as compared to SARS-CoV-1 virus. After SARS-CoV-2 binds to ACE2, there are two routes to enter the host cell, either by endocytosis or by membrane fusion [40].

There are two potential endocytic routes, the clathrin-dependent pathway and the caveolae (special type of lipid rafts containing caveolin-1)-dependent pathway [41,42]. The primary endocytic route for the coronavirus is considered the clathrin-dependent pathway [41]. However, the lipid raft-dependent endocytosis was described in SARS-CoV-2 infection as a novel pathway which is independent of clathrin and caveolae.

Moreover, SARS-CoV-2 uses TMPRSS2-mediated membrane fusion as a second way to enter host cells. In this case, ACE2 is involved in arresting and fixing the SARS-CoV-2 at the cell surface. Then, the TMPRSS2 induces a direct membrane fusion between the virus and the host cell [43]. Therefore, SARS-CoV-2 infection depends on the host cell factors ACE2 for entry and the host TMPRSS2 for SARS-CoV-2 spike (S) protein priming [44]. The surface spike glycoprotein of the virus (S-protein) constitutes a key element of host–viral interaction [3,45]. S-protein is formed of two domains separated by a protease cleavage site: S1 domain harboring the ACE2 recognition elements and the S2 domain for the fusion of membranes. A successful viral invasion involves a direct binding of S1 receptor-binding domain (RBD) of the virus to the ACE2 extracellular domain (PD) of the host, exposing the inter-domain, which is then cleaved by TMPRSS2, leading to virus–host membrane fusion [3,45]. The S1 RBDs from both SARS viruses (SARS-CoV-1 and SARS-CoV-2) have a similarity in overall architecture as they both interface with the same ACE2 surface, but differ, however, in the S-protein receptor binding motif, responsible for coronaviruses’ specificity as well as for the host range. A crystal structure analysis of ACE2 suggested the presence of several hinge regions and N-glycosylations (including N90 glycosylation), also considered important for SARS-CoV-2 binding [46].

After binding to the virus, the expression of the ACE2 receptor is downregulated [33]. This decrease in ACE2 bioavailability as well as the Ang II-AT1R axis’ up-regulation enhance COVID-19-induced inflammation and pulmonary injury [36], and promote organ injury [33].

### 2.2. RAS Imbalance and Underlying Pathologies

Since SARS-CoV-2 uses ACE2 as a key receptor for cellular entry, the latter subsequent downregulation will decrease Ang II degradation, leading therefore to its accumulation [17,33]. This contributes to a potential dysregulation and overactivation of RAS (Figure 1) [17,47]. Studies have shown that RAS imbalance worsens COVID-19 prognosis and aggravates its pathogenesis [17,46]. Therefore, the most common underlying pathophysiology of COVID-19 is a viral acute respiratory distress syndrome (ARDS) coupled with the cytokine storm syndrome [17].

According to current knowledge, the main cause of death in COVID-19 patients in intensive care unit (ICU) is the ARDS secondary to SARS-CoV-2 pneumonia [35]. ARDS is characterized by a hypoxemia with increased capillary–alveolar permeability, reduced lung compliance, alveolar epithelial cell loss, neutrophil infiltration and a diffuse bilateral pulmonary infiltrate that could lead to alveolar and interstitial remodeling and fibrosis [48]. This could lead to the loss of pulmonary perfusion regulation and hypoxic vasoconstriction, as well as a low ventilation perfusion ratio [2], thus requiring mechanical ventilation [48]. According to Richardson et al., 80% of patients who required mechanical ventilation after COVID-19 infection evolved to death, emphasizing that ARDS is an underlying pathophysiology in COVID-19 patients which may be responsible for the high mortality rates [49]. It has been shown that RAS imbalance may influence the pathogenesis of ARDS through Ang II and bradykinin [48]. Experiments have revealed that rats with knock-out ACE2 exposed to non-SARS lung damage (such as endotoxin) developed a severe ARDS compared to the wild type rats [50]. Therefore, ACE2 was shown to have a protective effect in rat models of acute lung injury, with ACE, Ang II and AT1R being considered as lung injury-promoting elements [50]. Imai et al., have shown an upregulation of Ang II by ACE in the pathogenesis of acute lung injury through the AT1a receptor, leading therefore to severe lung failure [28]. A study conducted by Kuba et al., showed that the blockage of ACE2 or its genetic manipulation leads to increased lung edema, vascular permeability and neutrophil accumulation [50].

On the other hand, the cytokine storm syndrome due to inflammatory cytokine could be responsible for worsening the SARS-CoV-2 disease severity and its clinical outcomes [33,51]. The cytokine storm, due to ACE2 downregulation by SARS-CoV-2, favors a pro-inflammatory environment which is relatively related to severe tissue damages, contributing to ARDS and fatal outcomes in COVID-19 patients [12]. The increased amount of pro-inflammatory cytokines such as interleukin-1B (IL-1B), interleukin-6 (IL-6), interleukin-12 (IL-12) and interferon gamma (IFN-γ) in the serum was shown to be associated with pulmonary inflammation and extensive lung damage in SARS patients [51]. This leads to RAS imbalance along with the downregulation of pulmonary ACE2 and the upregulation of the ACE-Ang II-T1R axis, which could worsen the systemic inflammation, causing ARDS, multi organ failure and death [52]. Huang et al., noted that patients infected with COVID-19 had high levels of IL-1B and IFN-γ, while ICU patients had higher concentrations of granulocyte colony-stimulating factor (G-CSF), interferon gamma-induced protein 10 (IP-10), monocyte chemoattractant protein-1 (MCP-1), macrophage inflammatory protein-1 alpha MIP-1a and tumor necrosis factor alpha (TNF-α) than those who did not require ICU, suggesting that the cytokine storm was associated with disease severity [51]. The hyper-inflammatory state, where IL-6 and C-reactive protein (CRP) levels are raised, has been shown to predict severity of disease in some COVID-19 patients [33].

ACE2 receptors are widely distributed throughout the human tissues, but with different expression rates: (1) high in the heart, small intestine, kidneys, testes, adipose tissue and thyroid; (2) medium in the lungs, liver, colon, bladder and adrenal glands; and (3) relatively low in the spleen, blood vessels, bone marrow, muscle and brain [53]. Therefore, ACE2-expressing organs are not equally involved in COVID-19 pathophysiology, highlighting that other molecular determinants participate in cellular infection leading to multiple organ injury, which is a major COVID-19 manifestation in most critically ill patients [54]. Rysz et al., showed that the blockade of ACE2 or the infusion of Ang II in swine resulted in increased pulmonary artery pressure and coagulation, reduced blood oxygenation, abnormal lung perfusion, induction of a diffused alveolar damage and acute tubular necrosis. By comparing these observations to those seen in COVID-19 patients, they proposed that COVID-19 severity could partially be due to RAS imbalance [17]. Indeed, the imbalance of Ang II-ACE2-Ang-(1–7) axis has been shown to be implicated in human pulmonary arterial hypertension and its severity [55], as well as in the thrombo-inflammatory process [56]. Moreover, RAS imbalance was shown to be involved in cardiovascular diseases (CVD), such as congestive heart failure (CHF; otherwise known as congestive heart failure or heart failure), myocardial infarction and hypertrophy, and cardiomyopathy [57]. Zhang et al., have shown that the overexpression of Ang-(1–7) and ACE2 resulted in anti-atherosclerotic and protective effects of endothelial cell function, as well as an increased antioxidant ability and inhibition of the inflammatory response [30]. Moreover, Ang-(1–7), through the counterbalancing of Ang II physiological actions, exhibited protective effects in kidney and cardiovascular diseases [27]. In fact, Feng et al., observed an increase in cardiac myocyte diameters and vascular collagen following infusion of Ang II in mice. In addition, they noticed a protective effect of ACE2, which was involved in the reduction in Ang II-mediated hypertension and sympathetic nerve activity [27]. Lovren et al., have shown an impaired endothelium-dependent relaxation in ACE2-deficient mice. However, the overexpression of ACE2 in human endothelial cells stimulated endothelial cell migration, limited monocyte and cellular adhesion molecule expression and attenuated Ang II-induced production of reactive oxygen species (ROS) [32]. This study also reported that *ACE2* gene transfer represents a protective mechanism in the endothelium by increasing antioxidant ability. Liu et al., have detected an increased Ang II level rate in COVID-19 patients, which seemed to affect the disease severity and to have led to immune disorder. Indeed, Ang II was significantly higher in critically ill patients than those with mild illness, and an abnormal rate of blood lymphocyte was relatively higher in patients with elevated seric Ang II concentrations [33].

Although the main COVID-19 feature is the respiratory compromise, studies have shown that the coagulation phenomenon, also associated with COVID-19, is manifested by an elevated D-dimer level, the presence of fibrin thrombi within capillaries and vessels [58]. The majority of recent studies have underlined the high thromboembolism risk in SARS-CoV-2 patients, especially in those with severe COVID-19 pneumonia [56]. Laboratory findings have shown the development of disseminated intravascular coagulation (DIC) in 70% of patients [58]. Tang et al., have described, retrospectively, the coagulation feature and its outcome in 183 consecutive patients with novel coronavirus pneumonia. The overall mortality rate was 11.5%, and non-survivors revealed significantly high levels of fibrin degradation product and D-dimer, along with a longer prothrombin time compared to survivors. In addition, DIC appeared in most of the deaths [59]. Several other mechanisms have been considered to be involved in this pathology, including deregulation of the immune response, complement and coagulation cascade activation and endothelial cell inflammation. However, the imbalance of RAS may have an essential function in the thrombo-inflammatory mechanism. The Ang II/AT1 axis activation may be the catalyst for initiating the coagulation cascade in some patients [33]. In addition, the loss of ACE2 and the upregulation of Ang II lead to endovascular thrombosis, vasoconstriction, endothelial injury and increased blood volume and, thus, DIC [56]. The DIC results from the cytokine release and the activation of monocytes and endothelial cells, with the secretion of Von Willebrand factor and expression of tissue factor. In addition, the thrombin free circulation, uncontrolled by the natural anticoagulants, can stimulate fibrinolysis and activate the platelets [59].

Furthermore, since the ACE2 receptor is found expressed at the surface of many cells including the cerebral neurons, neurological disorders and cognitive behaviors are also observed in COVID-19 patients [60]. These disorders include dysgeusia, anosmia, headache, delirium, seizures, encephalitis and stroke [12]. These signs are related to the overactivated RAS and the nitric oxide (NO) levels in the brain. In fact, the effect of Ang II on the vasoconstrictor AT1R diminishes the NO production, leading to a decrease in its concentrations [60].

CHF is a multi-factorial disease process that may develop over a long period of time and is characterized by over activation of the RAS and the sympathetic nervous system, resulting from various insults to the myocardium, the most common of which is ischemic heart disease [61,62]. Furthermore, there is convincing evidence that inflammation is associated with an increased CV risk independently of traditional risk factors [63]. However, scientists, and especially cardiologists have not yet reached a consensus on the implication of the SARS-CoV-2 induced cytokine storm in the development of post-COVID-19 CHF. In CHF, activation of both the innate and adaptive immune system occurs due to the higher activation and expression of toll-like receptors (TLRs), in particular TLR-2 and TLR-4 that, independent from prevalent systolic or diastolic dysfunction, result in the production and release of proinflammatory cytokines such as TNF-α, IL-1 and IL-6, activation of the complement system and production of autoantibodies [63]. This inflammatory status, probably driven by the coexistence of traditional CV risk factors, is burdened with the activation of some neurohormonal systems, such as the RAS, which increases both cardiac post-load and cardiac work and negatively impacts cardiac function deterioration [64]. Patients with CHF are also particularly vulnerable to the development of venous thromboembolism and its related complications of pulmonary embolism and right ventricular failure [65]. In line with this, CHF-associated comorbidities, such as chronic obstructive pulmonary disease, hypertension, obesity, type-2 diabetes mellitus, atherosclerosis, etc., are all characterized by mild inflammation and increased oxidative stress [66,67]. As described in Figure 1, SARS-CoV-2 infection can lead to disruption of the protective arm of the RAS and induces a more serious COVID-19 disease in ~20% of patients, which is often driven by an uncontrolled excessive immune response termed as a cytokine storm. In most cases, these patients develop rapid onset of pneumonia, which can lead to respiratory failure and ARDS. Many patients also experience extra-pulmonary manifestations affecting heart, kidneys, brain and other organs with multi-organ failure in the worst case. These complications are not only a result of direct SARS-CoV-2 infection, but also of a systemic endotheliitis, causing coagulopathy and thromboembolic complications.

### 2.3. RAS Component Polymorphism

Many studies described the association of the RAS component genetic variation with the prevalence of COVID-19 diseases [47,68]. Therefore, it has been thought that genetic factors may render the host resistant or susceptible to infection with SARS-CoV-2 [47]. The prevalence and disease outcome were linked to *ACE* polymorphisms [36]. The genetic polymorphisms of *ACE1* and *ACE2* genes may change their levels of expression, therefore leading to an increase in capillary permeability, coagulation, fibrosis and apoptosis in alveolar cells [69]. In a pilot study conducted by Cafiero et al., it was found that some genetic variants in the RAS pathway may be potential actors for determining the clinical outcome and the pathological conditions associated COVID-19, such as DIC, interstitial pneumonia, thrombosis, conjunctivitis and the cytokine storm [35]. Thus, inflammation and lung injury caused by ACE2 decrease, following viral binding, could be negatively affected by *ACE*’s different genotypes, which in turn could increase ACE expression levels and then those of Ang II [36]. The knowledge of these polymorphisms could help the management of COVID-19 infected patients [36]. The major RAS component polymorphisms are illustrated in Table 1.

#### 2.3.1. ACE1 Polymorphism

The human *ACE1* gene is located on the long arm of chromosome 17 (17q23.3) [35,47,70] and contains 25 introns and 26 exons [70]. This gene possesses an insertion (I) or deletion (D) of a 287-base pair (bp) *Alu* repeat sequence within intron 16 [35,47,70] which could lead to a lower or higher ACE serum level, respectively [35]. Therefore, in *I*/*D* polymorphism, three different genotypes are possible: *I*/*I*, *I*/*D* and *D*/*D* [47]. The mean ACE activity levels in *D*/*D* genotype individuals were approximately twice those in *I*/*I* genotype individuals [71]. Since ACE and ACE2 levels are related to each other, it was suggested that lower levels of ACE2 could be expected in patients possessing the *D* allele of *ACE I*/*D* polymorphism [70]. Indeed, it has been shown that some variants of the *ACE* gene, such as *ACE I*/*D* polymorphism, affect both the activity and the level of ACE, which could render the patient susceptible to different COVID-19-related illnesses, including cardiovascular, kidney and autoimmune diseases, hypertension, type 2 diabetes and overweight [36]. Yamamoto et al., suggested that *ACE1 I*/*D* polymorphism could be a potential genetic marker for COVID-19 infectivity and may be involved in the various diseases caused by the virus, including the cytokine storm, pneumonia, thrombosis, DIC, ischemic stroke and renal injury [47]. In addition, the *D* allele is associated with a greater risk of diabetes mellitus, diabetic nephropathy, hypercoagulability, hypertension, endothelial damage, obesity and cerebral ischemia [72]. Yamamoto et al., found a significant negative correlation between the number of COVID-19 cases and deaths and the *ACE1* *I*/*I* genotype frequency among European and Asian populations. In other words, the increase in the *ACE I*/*I* genotype decreased SARS-CoV-2 death [47]. On the contrary, Pati et al., have shown a significant positive correlation between the *D* allele frequency in Asian populations and the number of COVID-19 infected cases/million [70]. Cafiero et al. have conducted a pilot study to determine the frequencies of six polymorphisms in the RAS system, including the *ACE1* gene (rs1799752), within symptomatic and asymptomatic patients affected by COVID-19. They have indicated that the *I* allele and the genotype *I*/*I* are significantly less frequent among the symptomatic than the asymptomatic patient group [35]. A meta-analysis performed by Nie et al., to determine the association between the risk of pneumonia and the *ACE I*/*D* polymorphism revealed that *D*/*D* genotype carriers had a 53% increased risk of developing pneumonia compared to those with *I*/*I* and *I*/*D* [71].

*ACE I*/*D* polymorphism may be an important prognostic factor for ARDS outcome [73,74], and could play a potential role in the pathogenesis of ARDS [73]. In fact, it was shown that the risk of morbidity and mortality from ARDS is higher in patients with the *D*/*D* genotype of *ACE1* [48]. The prospective study performed by Adamzik et al., on Germans of Caucasian ethnicity with ARDS showed that homozygous *D*/*D* genotype patients were at higher risk for mortality (fivefold) and death compared with the *I*/*I* genotype [48]. In addition, Jerng et al., showed that the mortality rates are significantly different among ARDS patients with *ACE I*/*D* genotypes (75%, 65% and 42% for *D*/*D*, *I*/*D*, and *I*/*I*, *I*/*D* respectively) [75]. Therefore, *ACE I*/*I* genotype patients have a significantly better survival chance than those with the other genotypes [73,74]. Moreover, Marshall et al., provided the first evidence of a genetic influence of ACE, and particularly the *D* allele, in the development and progression of ARDS [73].

Furthermore, *ACE* polymorphism might represent a genetic risk factor to thromboembolism in COVID-19 patients. Calabrese et al., and. have noticed a significantly higher *ACE D*/*D* polymorphism prevalence in patients compared to pulmonary embolism negative ones [56]. However, the *I*/*D* genotype was significantly lower in pulmonary embolism positive patients [56]. Studies have also shown an association between the *G*/*G* genotype of rs4343 polymorphism of *ACE* and the high circulating levels and activity of ACE receptors [36]. Íñiguez et al., have investigated whether the rs4343 and rs4341 polymorphisms of the *ACE* gene could explain the different outcomes of COVID-19 patients. Results showed that the *G* allele of rs4341 and rs4343 confers an additional risk in COVID-19 prognosis, and was related to COVID-19 severity in hypertensive patients and to a higher severity level in patients with dyslipidemia [36].

It would also seem that the racial variance of *ACE I*/*D* genotype accounts for the different prevalence and outcomes due to COVID-19 [56]. In fact, the *D* allele’s higher frequency observed in the African American population was correlated with higher mortality rates, as compared to white people and Indians (89% for African American and 69% to white people and Indians) [76]. Pati et al., have shown a lower *D* allele frequency in Eastern Asian populations, such as China and Japan (38.42 and 37.39, respectively) compared to Middle Eastern populations, such as in Lebanon and Palestine (69.61 and 74.27, respectively) [70]. Likewise, populations of Italy, Spain and France have *D* allele frequencies reaching 87%. In contrast, in Eastern Asian populations (Korean, Chinese, Taiwanese and Japanese) a higher frequency of *ACE I*/*I* genotype was recorded compared to European populations (33% to 51% versus 13% to 27%), which could explain the high COVID-19 fatality in European patients (especially among Spanish, Italian and French) [77]. These findings also conformed to those found by Hatami et al., in which a higher frequency of the *I* allele was observed in China and Korea, while in European populations such as Germany, Italy and France, a higher frequency of the *D* allele was recorded [68].

#### 2.3.2. ACE2 Polymorphism

The analysis of ACE2 protein altering variants and polymorphisms is important to understand the consequent changes made to the molecule, hence allowing the elucidation of population risk profiles. Suryamohan et al., found a total of 298 unique protein altering variants across 256 codons distributed throughout the 805 amino acid long human ACE2s [3]. The *ACE2* gene, which forms approximately 40 kb of genomic DNA, is located on the chromosome X (Xp22.2) and contains 18 exons. It shares 40% homology of the amino acid sequence with ACE1 at its N- and C-terminal domains [79]. Some genetic variations in *ACE2* could alter the viral cellular entry, either by increasing the SARS-CoV-2 binding affinity or by changing its expression levels. Researchers have shown particular interest and attention to 14 SNPs of *ACE2*, including rs233574, rs2285666, rs2158083, rs2074192, rs2106809, rs4830542, rs4646188, rs4240157, rs879922, rs1514282, rs1514283, rs4646155, rs4646176 and rs1978124 [79].

Among others, the K26R ACE2 altering variant could potentially decrease or increase the ACE2/S-protein binding affinity and thus alter the viral ability to infect host cells. Moreover, the ACE2 C-terminal collectrin-like domain variant (N720D) affects the TMPRSS2–ACE2 complex and favors TMPRSS2 binding as well as its cleavage, thus helping it bind to S-protein and promoting viral entry [77]. It has been shown by molecular docking simulations that six ACE2 variants (T55A, E75G, I21T, K26R, A25T and E37K) increased the binding affinity of ACE2 to S-protein RBD, while 11 other variants (I21V, K26E, M82I, E35K, T27A, Y50F, N51D, S43R, K68E, E23K, and N58H) decreased it [87]. A study has also elucidated a significantly lower *T* allele frequency in rs2074192 SNP in an asymptomatic group compared to a symptomatic one, in both women and men [35].

It is well established that the *T* allele of rs2074192 polymorphism is associated with CVD, retinopathy in type-2 diabetes mellitus, hypertension and hypertensive left ventricular hypertrophy [88,89]. Therefore, the contribution of the *T* allele to more severe outcomes of SARS-CoV-2 infection is now uncontroversial, with a large amount of evidence supporting this relationship [35,90].

Hamet et al., studied the relation between the *ACE2* gene and hypertension (HT) in correlation with pre-existing factors related to COVID-19 severity in the French-Canadian population. It has been shown that the *T* allele of the rs2074192 SNP of *ACE2* gene was a risk factor for HT in adult obese males, and even more in smokers, compared to non-obese and non-smoker males and females. In the same study, it was revealed that this SNP, located in intron 16, could modify the binding of some regulatory factors, including ARID5A, which is an RNA-binding protein stimulated by inflammation and possessing a role in post-transcriptional events [90]. Also, Suryamohan et al., have examined human multiple ACE2 variants that will either protect individuals from SARS-CoV-2 or render them more susceptible to the virus. Using reported structural data, they showed that the following variants: D38V, H34R, K68E, F72V, K31R, Y50F, N33I, E35K, E37K, N51S, M62V, Y83H, G352V, Q388L, D355N, G326E and D509Y, protected individuals by decreasing binding to the S-protein. However, T27A, N64K, I21V, S19P, E23K, K26R, T92I, H 378R and Q102P increased susceptibility to the virus for their ability to favor the viral entry. K26R mutation, for example, has been shown to weaken the linked glycan (N90) coordination, interfering with its capability to protect hosts from the viral infection. In addition, T92I and K26R variants showed increased affinity for S-protein, in contrast to E37K and K31R, compared to the wild-type ACE2 [3]. Devaux et al., have also shown that species, including *Homo sapiens*, carrying a sequence with K353, N90, K31 and Y41 in their ACE2, are more likely to be susceptible to SARS-CoV-2 infection, while others were less susceptible or considered resistant [46]. Hou et al., demonstrated by a comparative genetic approach that ACE2 or TMPRSS2 DNA polymorphisms were likely associated with genetic susceptibility to COVID-19. In fact, by altering angiotensinogen-ACE2 interactions, they found that the p.Arg514Gly variant of *ACE2* in the African/African-American population is related to pulmonary and cardiovascular conditions. As for the p.Val160Met (rs12329760) polymorphism in TMPRSS2, it provides potential explanations for differences in COVID-19 genetic susceptibility as well as for risk factors, including high risk male and cancerous patients [44]. Pouladi and Abdolahi (2021) showed that rs4646188, rs233574 and rs2074192 were able to cause modification of the *ACE2* RNA secondary structure. These variations may deregulate *ACE2* transcription/translation and protein stability, thereby altering viral binding to ACE2 and pathogenesis. In fact, modification of the mRNA secondary structure may expose the sensitive amino acid sequence of the protein or manipulate its proper folding, causing it to be more susceptible to proteases. Such variants could therefore lead the way to differences in SARS-CoV-2 susceptibility among individuals [79]. Guo et al., used the Genome Aggregation Database to systemically characterize missense variants in *ACE2* gene, and then studied the structural flexibility of ACE2 and its interaction with SARS-CoV-2 S-protein, which could affect the viral recognition and infection. They identified 12 ACE2 deleterious missense variants, in which the p.His378Arg has been shown to weaken and decrease ACE2 activity and the p.Ser19Pro may modify the most important helix to the S-protein. In addition, it has been shown that seven missense variants (i.e., p.Asp206Gly, p.Gly211Arg, p.Arg219His, p.Arg219Cys, p.Ile468Val, p.Lys341Arg and p.Ser547Cys) may affect the protein’s secondary structures [16].

#### 2.3.3. AGT Polymorphism

The *AGT* gene, located on chromosome 1q42.2, encodes for the angiotensinogen precursor, which is expressed in the liver. In response to lowered blood pressure, AGT is transformed by renin into Ang I. The latter is then cleaved by the ACE into Ang II, which is a physiologically active form playing a major role in electrolyte homeostasis and in blood pressure stability [35]. So far, no data of *AGT* polymorphism are available to explain its effects in COVID-19 patients. However, much experimental evidence suggested that some genetic sequence variants in AGT could be related to the development of CVD, hypertension and blood pressure [35]. Since the novel coronavirus was associated with these pathologies in some patients, it would therefore not be irrational to link *AGT* polymorphism to COVID-19 disease.

Two main *AGT* gene polymorphisms due to base substitutions were studied and shown to be associated with a high risk of developing various cardiovascular diseases: M235T, which is the substitution of methionine by threonine at codon 235, and T174M, which is the substitution of threonine by methionine at codon 174 [83]. In addition, other *AGT* gene polymorphisms have been shown to be linked to hypertension, including G217A (substitution of guanine by adenosine at codon 217) and A20C (substitution of adenosine by cytosine at codon 20) [91,92]. The A20C and G217A genotypes, located in the promoter region of the *AGT* gene, have been shown to influence *AGT* transcriptional activity and, therefore, the AGT serum levels [91,92]. It has also been reported that the M235T polymorphism is related to increased AGT levels in serum [57,81].

Tran et al., have shown that Vietnamese patients diagnosed with essential hypertension, and having the T/T genotype of AGT M235T, had a significantly greater left ventricular mass index compared to those having the heterozygous M/T genotype [81]. Moreover, Imen et al., concluded that AGT M235T polymorphism in a Tunisian population could be associated with increased risk of heart failure and death. In fact, they noticed that the T/T genotype of AGT M235T was more frequent in the heart failure group compared to the control (58% vs. 22%, *p* < 0.001). However, the normal genotype of ACE (M/M) was detected more frequently among the non-HF group (44% vs. 15%, *p* < 0.001). The death rate was also higher in the heart failure group for patients having the *T*/*T* genotype compared to those with the *M*/*M* genotype (58% vs. 13%, respectively) [80]. Raygan et al., have demonstrated a relation between AGE M235T polymorphism and the risk of myocardial infarction in the Asian population. Indeed, a significant association was noted between myocardial infarction risk and the *T*/*T* genotype (odds ratio [OR] 2.08, 95% confidence interval [CI] 1.08–4.00, *p* = 0.029), specifically with the *T* allele (OR 1.45, 95% CI 1.06–1.99, *p* = 0.021) [82]. In addition, an *in silico* analysis showed that M235T fundamentally changed the function of AGT [82]. Rani et al., have examined the association of AGT with the risk of cardiomyopathy in Asian Indian patients. They revealed a significant prevalence of the T/T genotype of AGT (M235T) in three groups of patients (hypertrophic cardiomyopathy (HCM), dilated cardiomyopathy (DCM) and restrictive cardiomyopathy (RCM)) compared to the control group, indicating that the *T*/*T* genotype was significantly associated with enhanced risk of the disease in HCM, DCM and RCM [78]. Wang et al., have demonstrated a significant association of ACE M235T polymorphism and the development of atrial fibrillation (AF) in Asians [57]. Recently, Cafiero et al., have identified that rs699 SNP of *AGT* gene could be a potent tool for predicting and understanding the outcome of COVID-19 patients. Indeed, the rs699 SNP showed a significant increase in *T* allele frequency compared to the total population from the GnomAD and 1000 Genome Project [35]. Many studies also pointed out a significant association between the risk of developing myocardial infarction and coronary artery and the T174M polymorphism of *AGT* gene in Caucasian and Asian populations [93,94]. Finally, both the A20C and G217A AGT polymorphisms were significantly linked to the risk of essential hypertension [91,92].

#### 2.3.4. AT1R and AT2R Polymorphisms

There are two types of Ang II receptors: type 1 receptor (AT1R) and type 2 receptor (AT2R), encoded by two separate genes [95]. The human *AT1R* gene is located on the long arm of chromosome 3 (3q21–q25) and is formed of four introns and five exons with a >55 kb length [84,85]. The first four exons constitute the 5′ UTR region, whereas the fifth exon contains the coding region [85]. ATR1 is expressed in many organs such as the lungs, heart and brain, and has been demonstrated to have the most cardiovascular effects of Ang II [84]. The *AT1R* gene is polymorphic, and several variants have been discovered [86]. Five SNPs have been identified in the *AT1R* gene, namely G1517T, A1166C and A1878G, located in the 3′ UTR, and T573C and A1062G, located in the coding region [85]. The most studied SNP is the A1166C (aka rs5186) variant (nucleotide substitution A to C at 1166 position), being located in the 3′UTR of *AT1R* gene (3q21eq25). This variant has been shown to be associated with systolic blood pressure, left ventricular hypertrophy, hypertension, aortic stiffness, myocardial infarction (MI), carotid intimal–medial thickening, coronary artery disease (CAD) and stroke [84,86]. The highest 1166C allele frequency was recorded in Caucasians (0.31), while the lowest (0.02) was observed in Africans [85]. The C1166 allele was also shown to decrease microRNA-155 binding to a specific sequence in the 3′UTR. Therefore, miR-155 will no longer efficiently attenuate the translation, thus leading to an increase in AT1R density [85].

The frequency of the *C* allele of *AT1R* was more elevated in a hypertensive group of patients compared to the control (39.4% vs. 25.9%) [96], whereas the A1166 variant seems to have a lesser susceptibility to hypertension [97]. Several studies showed that the *AT1R* gene is involved in the development of aortic stiffness in hypertensive patients. Another study reported that common genetic variation at the *AT1R* gene locus influences the risk of essential hypertension in the Finnish population [98,99].

On the other side, the *AT2R* gene, positioned on the sexual chromosome X, (Xq23–26) is highly expressed in fetal tissues, but its expression level decreases with age to be present in only a few adult organs. The AT2R plays an opposite function of the AT1R by stimulating vasodilatation, stimulating apoptosis and inhibiting cell proliferation. The common polymorphism of this receptor (rs1403543) is detected in the translation initiation site (−1332) [84].

Abd El-Aziz et al., recruited 132 Egyptian males with premature coronary artery disease (PCAD) and 132 control individuals in order to determine if *AT1R* and *AT2R* gene polymorphisms increase the susceptibility to develop PCAD. They showed that the *ATR2 G* allele and *ATR1 C*/*C* genotype increased PCAD risk by 1.3 and 2.9, respectively, and the susceptibility to metabolic syndrome by 2.3 and 4.5, respectively [84].

All of the above genetic variations in the RAS could explain the strong variability in the susceptibility to—and severity of—COVID-19. However, additional risk factors, including the role of adaptive and innate immunity, could also modify the disease outcomes [3]. More attention should therefore be given to predictive medicine, which might be a significant approach to minimize pandemic situations affecting the world, by preventing and treating diseases based on genetic predisposition of patients, their lifestyle and the environment. In this context, in silico evaluation of interactions between some pharmaco-genetically relevant SNPs and drug candidates for fighting COVID-19 was shown to be promising. This further strengthens the importance of the personalized approach (predictive medicine) in the development of strategies to counteract the complicated clinical scenarios of COVID-19 that are seriously affecting the world [35].

## 3. Correlation of Habits, Gender and COVID-19 with RAS Polymorphism

**Figure 2:**
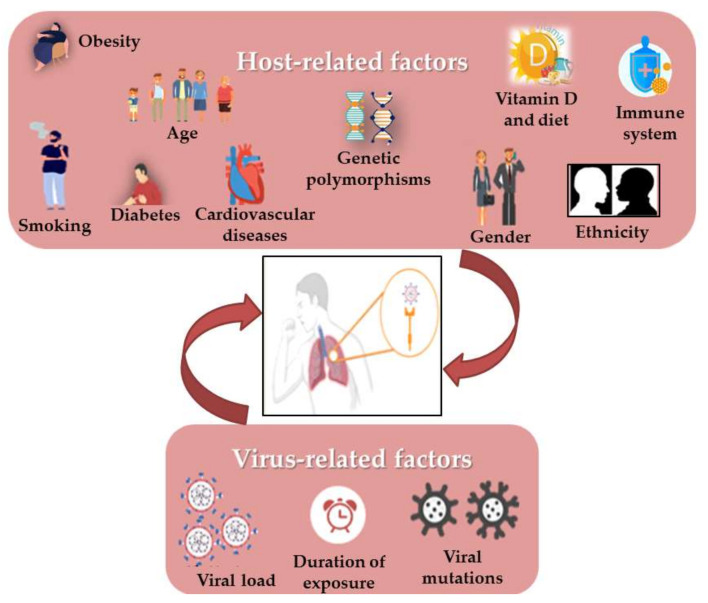
Host and virus-related factors affecting COVID-19 outcome. Several factors related to the virus and to the host could account for COVID-19’s severity among individuals. A strong immune response is essential to eliminate the virus before its progression to more severe stages; therefore, harmful behaviors such as sedentary lifestyle, obesity, elevated tobacco consumption and unhealthy diet may weaken the immune system and render the host more sensitive to the virus. Health status is yet another factor influencing the clinical manifestations of SARS-CoV2 infections. As such, people of older age, especially those with comorbidities such as diabetes and lung and cardiovascular diseases may experience a more severe disease outcome. Moreover, the risk seems to be higher in males than in females, possibly because of the hormonal differences between the two genders as well as their genetic background, especially in the RAS components. RAS overactivity has also been described in metabolic syndrome, type 2 diabetes and obesity, all high-risk conditions for COVID-19 infection and severe disease. On the other hand, a higher viral load of SARS-CoV-2 as well as a sufficient duration of exposure to the virus could be related to more severe illness and even death. More importantly, the viral genetic mutations may favor the appearance of more severe variants, which could have a higher infectivity rate as well as a higher fatality rate among sensitive populations.

Still now, it is surprising that some COVID-19 patients are asymptomatic while others have much more severe disease outcomes. Moreover, viral respiratory infections are generally more harmful in children than in adults, but this appeared to be inverted in SARS-CoV-2 infection. It has been shown that some virus-related factors (viral load in the inoculum, the exposure duration and viral genomic mutations) can influence the severity and outcome of the disease [100] (Figure 2). Similarly, it quickly became obvious that several risk factors such as age, gender, the presence of comorbidities (such as smoking, immune status, diabetes, cardiovascular disease including hypertension, respiratory diseases and cancer) and the genetic background seem to control the manifestations and outcome of infection [41,45,101,102]. In addition, it has also drawn attention to vitamin D deficiency [103], as well as the ethnic differences, such as the black and South Asian ethnicities, and the lower socioeconomic statuses which are considered to increase risks [54]. Most mortalities occurred in the elderly and in men compared to women (4.7% vs. 2.8%). Moreover, the death of patients with no pre-existing conditions is approximately 10 times lower than in those with pre-existing ones. As in the 2003 SARS epidemic, hypertension has the most comorbidity frequency in non-survivors, in addition to cardiovascular diseases, obesity and diabetes, especially in smokers [54,90]. There is evidence that the RAS upregulation in the adipose tissue may lead to hypertension and insulin resistance in obese people. [37] 

### 3.1. COVID-19 and Age

SARS-CoV-2 is believed to have a much higher transmission rate compared to MERS-CoV and SARS-CoV-1. However, in the three cases, low infection rate and a mild disease outcome were shown in children [104]. Age is an important factor in the outcome of viral respiratory infections. During the Spanish flu pandemic in 1918, the mean death rate was high in people aged younger than five years, 20–40 years old, and older than 65 years [105]. The majorities of COVID-19 infected people were of older age (ranging between 30 and 79 years, approximately) and many developed severe conditions and needed intensive care [104].

With other coronaviruses, such as SARS-CoV-1 and human coronavirus-NL63 (HCoV-NL63), it has been observed that children are relatively resistant to infection, similar to the current observations for SARS-CoV-2 [106].

Wu et al., have shown, in a retrospective cohort study on 201 COVID-19 patients with pneumonia, that older age (≥65 years old) and comorbidities (diabetes, hypertension) were significantly associated with higher risks of developing ARDS as well as a higher death risk after the viral infection [107]. Therefore, many arguments have been presented to explain the susceptibility of older age patients to SARS-CoV-2 infection and to develop more severe outcomes following the infection. The first explanation could be attributed to the immune system of younger groups, which could be more performant in resisting to infection or because of having a cross immunity due to a previous contact with other types of coronaviruses. Another argument could be the fact that by having predisposing chronic diseases such as diabetes and CVD, the elderly could be at higher risk. Another explanation worth mentioning could be the low expression level of ACE2, which could decrease transmission in young groups [104]. Xudong et al., have shown, in both gender rats, that ACE2 protein expression is significantly reduced with aging. In addition, they observed a significant decrease, with age, in the number of cells stained for ACE2 enzyme by immunohistochemical analysis [108]. The decrease in soluble ACE2 in SARS-CoV-2-infected patients favors Ang II upregulation, which is positively correlated to the viral load and multiple organ damage [108]. Sharif-Askari et al., have concluded that differences in COVID-19 severity between adults and children could be related to the differential expression levels of ACE2 receptors and TMPRSS2 in the airway epithelium. In fact, they found that children had a significantly lower ACE2 expression in the upper (nasal) and lower (bronchial) airways. In addition, the expression of ACE2 receptor and TMPRSS2 in the lung airway was found to be significantly upregulated in patients with COPD compared to healthy individuals [104]. Therefore, it can be hypothesized that ACE2 age-related expression might contribute to different pathological profiles in COVID-19.

Feng et al., have shown that a >75 years age group had a higher percentage of patients with critical diseases, comorbidities and death. In fact, the rate of COPD was shown to increase progressively with age. The 45-year-old age group also had higher IgM levels and lymphocyte counts [109]. Verma et al., have shown that an older age parameter (≥46 years) is significantly higher in patients with severe COVID-19 compared to mild cases (88.3% vs. 11.7%, respectively, *p* < 0.001) [69]. A possible explanation of the disease severity in the elderly is the increased presence of comorbidities and the decline in multiple areas of immune function with advancing age. Furthermore, the disease mortality at older age is related to hyper-inflammation and the development of the cytokine storm [103]. The study of Yang et al., showed that COVID-19 non-survivors were older compared with survivors, (64.6 vs. 51.9) and were more likely to develop chronic illnesses (53% vs. 20%). Indeed, older age patients (>65 years) have more chance to develop ARDS than younger ones [54].

On the other side, COVID-19 is capable of inducing more anxiety for older patients compared to younger ones. This anxiety is then transformed into higher stress profiles, which can have deleterious consequences on patient health [110]. A Chinese study showed that COVID-19 could lead to moderate or severe stress as well as to high anxiety levels. Wang et al., realized that COVID-19 anxiety was correlated with higher stress level in the totality of the sample. Moreover, in terms of response to stress, the anxiety was much worse in older adults. COVID-19-related stress shows important differences in risk and resilience for younger and older adults. Anxiety about developing COVID-19 was a stronger risk factor, but proactive coping was a stronger resilience factor for stress in older adults compared to younger ones [110]. Proactive coping is motivated by the belief in the rich potential of changes made to improve oneself and one’s environment [111]. Efforts are currently needed in order to reduce COVID-19 anxiety by increasing proactive coping in the elderly during this pandemic [110].

### 3.2. COVID-19 and Gender

The X and Y sex chromosomes display many levels of distinctions. Females possess two cell types in all their organs, each with one of the two X-chromosome being genetically active, while the other was permanently inactivated early during the embryo development [101]. X-chromosome inactivation is due to epigenetic developments that randomly pick and then permanently silence one of the X-chromosomes of women [101]. The higher incidence of premature coronary artery disease (CAD) in men is due to single point mutations in the chromosomal region of males (hemizygous for disease alleles) which are more likely to produce the effects of a recessively acting X-chromosome susceptibility gene than that of dizygotic women [84]. In fact, COVID-19 affects men more than women due to the higher concentration of ACE2 receptors in men, which is in turn also related to androgen levels [103]. The general tendency in females shows a better humoral and cellular immune response to the viral infection. Thus, women get rid of infections more rapidly and successfully than men.

The *I* allele of *ACE1* seems to be more present in women, while the *D* allele (related to a high ACE1 level) seems to have higher levels in men [112]. This significant gender-related difference in levels of ACE1, being lower in healthy and pathological females, suggests a higher ACE1/ACE2 imbalance in males during SARS-CoV-2 [112]. The Online Mendelian Inheritance in Man shows that the *ACE1* gene may be correlated with the virus’ progression, with the *D* allele having a genetic tendency for inducing the progression from pneumonia to SARS [101].

### 3.3. COVID-19 and Smoking

Smokers were considered as strongly susceptible to COVID-19. In fact, cigarette smokers are highly associated with adverse outcomes of COVID-19 and more likely to require clinical intervention [113]. Smoke is considered to have different components, including nitrogen oxides, phosgene, aldehydes, hydrogen chloride, ammonia and sulfur dioxide. Exposure to smoke impairs the airway mucosal function by causing protein denaturation, inflammation, injury of cilia, reduction in alveolar macrophages and damage to the capillary membranes, which leads to pulmonary edema [114].

Many data have shown a strong correlation between smoking, RAS and the outcome of COVID-19 infection. ACE2 expressed in lung cells could act as an interferon-stimulated gene, indicating that COVID-19 infection may lead to a positive feedback loop, helping the viral dissemination by increasing the level of ACE2 [113]. Li et al., have indicated that long-term smoking may be a risk factor for COVID-19. Indeed, they have shown an up- regulation of *ACE2* gene expression in long-term smokers. In addition, after analyzing ACE2 in SARS-CoV-2 infected cells, they suggested that ACE2 is also involved in the post-infection pathology, including viral genome replication, immune response and cytokine secretion [115]. Yilin et al., have demonstrated that the protein level of ACE and ACE2 increased at 1 h and 1–4 h, respectively, after smoke inhalation in the lung tissue of rats. They have concluded that the RAS could participate in lung injury due to the up-regulation of ACE, which would lead to an increase in Ang II in the lungs. The latter accumulation in lungs causes pulmonary vasoconstriction, promotes monocytes/macrophages infiltration and enhances the secretion of the transforming growth factor-1 (TGF-1), leading to pulmonary hypertension and fibrosis formation, and hence to a more severe lung injury [114]. Similarly, Cai et al., have observed that ever-smokers had a significant up-regulation of *ACE2* gene expression in lungs (by 25%) in comparison to non-smokers. Such an increased expression level upon smoking was believed to enhance the risk for COVID-19’s binding and entry into smoker’s lungs. In addition, they concluded that, based on ACE2 expression, smokers are at risk of developing COVID-19 infection complications which could contribute to variations in infection susceptibility, disease severity and treatment outcome [116]. Smith et al., have also demonstrated that smoking causes an upregulation of ACE2, both in human and rodent lungs, in a dose-dependent mechanism. They proved, however, that smoking reduction decreased ACE2 expression levels by reducing the number of lung secretory cells [113].

Some studies documented a strong relationship between smoking and the RAS polymorphism. Smoking and RAS components (in pathological conditions) have been shown to favor NO degradation, and thus free radical production, causing endothelial injury and impaired vasodilatation [90]. In the Rotterdam prospective cohort study conducted by Arias-Vásquez et al., on 6869 elderly participants, the *ACE* D/D genotype carriers were shown to have an increased mortality risk at an early age (below 65 years). However, this association was significant only in smokers, suggesting a possible interaction between the *ACE* gene polymorphism and smoking. They have suggested that early mortality due to ACE and smoking interaction could be a smoking-dependent effect of ACE on vascular homeostasis. In fact, the synergetic effect of the *D* allele and smoking could lead to atherosclerosis and coronary artery disease [117]. Recently, Hamet et al., (2021) showed that the *T* allele of SNP rs2074192 of the *ACE2* gene predisposes to hypertension earlier onset in overweight French-Canadian male smokers [90]. Therefore, smoking could be a risk factor for COVID-19 by affecting ACE2 expression, and may provide valuable information for identifying and stratifying more susceptible populations.

### 3.4. COVID-19 and the Cardiovascular System

CVD, including hypertension, coronary artery disease (CAD) and heart failure, is common in older patients [118], which may increase COVID-19 infection risk and enhance the disease’s progress [118,119]. It has been proven that about 50% of the variability of the major risk factors for coronary heart disease (CHD) is genetic [120]. SARS-CoV infected patients often suffer from cardiac diseases as well as sudden death [121]. Amara et al., have shown that several risk factors had significant effects on CAD, including diabetes and dyslipidemia [122], in addition to gender, age, family history, smoking and genetic factors [123,124].

The RAS is considered as one of the major regulators of cardiovascular physiology by controlling sodium homeostasis, cardiovascular remodeling, maintenance of vascular tone [122] and the regulation the blood pressure [123]. Ang II, the active component of the RAS, plays a major role in the regulation of cardiovascular homeostasis, and regulates hypertrophy, vasoconstriction, migration and proliferation of vasculature smooth muscle cells. Furthermore, AT1R has been implicated in the pathogenesis of arteriosclerosis and hypertension [125]. In addition, the loss of ACE2 could favor the development of cardiomyopathy, pulmonary injuries and kidney disease [121]. Oudit et al., have shown in a murine model that SARS-COV2 has downregulated the *ACE2* mRNA expression in the heart, and thus has led to myocardial dysfunction and inflammation. The myocardial damage was proven by macrophage-specific staining in which an increase in myocardial macrophage infiltration was shown [121]. In addition, the same study revealed that SARS-CoV is able to infect the myocardium, since it was detected in the heart of 35% of those subjects who died considerably earlier [121]. Since the expression of ACE2 in the heart is higher than that in the lungs, the main target of the virus, the heart might then be highly vulnerable and at higher risk of damage following the infection with the virus. Furthermore, Chen et al., have found that pericytes, which are vascular mural cells of the blood microvessel basement membrane, have a high ACE2 expression, rendering them a potential host cell for SARS-CoV-2. The pericyte infection may result in microvascular dysfunction. It was also shown that COVID-19 patients with previous heart disease have an increased ACE2 protein and mRNA expression, and could progress to severe and critical cases, including death [119]. Xie et al., showed that CVD plays a major role in COVID-19 severity, since the rates of CAD and hypertension in severe groups were significantly higher than those in non-severe cases. In addition, it has been reported that IL-6, high-density lipoprotein (HDL) cholesterol, creatinine, C-reactive protein, high-sensitivity cardiac troponin I (hs-cTnI), D-dimer levels and prothrombin time were significantly higher in the severe COVID-19 CVD group than those in the non-severe one [118]. Wallentin et al., have shown a correlation between a higher level of soluble ACE2 and cardiovascular diseases, as well as diabetes. In fact, higher levels of cardiovascular biomarkers including GDF-15 (growth differentiation factor) and NT-proBNP (N-terminal probrain natriuretic peptide), related to the high level of ACE2 and a higher risk for mortality and cardiovascular disease, might be prognosis factors to identify severe COVID-19 infections [126]. NT-proBNP is considered an indicator of myocardial necrosis and dysfunction, whereas GDF-15 is an unspecific indicator of inflammation, cellular stress and biological aging, which is elevated in cardiovascular diseases as well as in other diseases such as diabetes mellitus and renal diseases [126].

The RAS plays a role in cardiovascular homeostasis and hemodynamics, the reason for which is the special interest that has been accorded to its role in developing CVD [120], mostly due to some allelic variants in gene encoding for the RAS components [122,127]. Some mutations in these genes might influence the initiation and the outcome of CAD [124]. Essential hypertension, for example, is related to some genetic mutations in RAS components, such as the *AT1R* gene, and to angiotensinogen (AGT) M235T and *ACE I*/*D* polymorphisms [38].

Some of CVD’s traditional risk factors, such as cigarette smoking and hypercholesterolemia, could influence RAS function, including the AT1R [125]. Others, such as obesity, diabetes mellitus and family history of CVD at a young age, stimulate ACE synthesis, which leads to an excessive vasoconstriction, enhancing the ischemic risk [124]. Therefore, identifying the associations of genotypes with phenotypes would be an important tool in cardiac diseases [124]. Several studies have shown that RAS component polymorphisms in some individuals are essential in the development and progression of CAD, specifically the *ACE I*/*D* polymorphisms [122]. The latter also plays an important role in the pathogenesis of atherosclerosis and has been implicated in endothelial function [120,122]. The *ACE I*/*D* polymorphism and its correlation with CVD and mortality has been extensively investigated [113]. The *D* allele has been consistently related to higher ACE levels, predicting a higher risk of cardiovascular disease. Some previous studies have shown that the *ACE D*/*D* genotype is associated with the development of myocardial infarction and left ventricular hypertrophy [120]. Carriers of the *ACE D* allele possess a higher tissue ACE mRNA expression, an increased circulating ACE concentration and Ang II, as well as an increased bradykinin degradation. These observations affect cellular growth and proliferation by stimulating various growth factors and cytokines, and could impair endothelium-dependent vasodilatation by reducing NO bioavailability, which could damage vessels leading to hypertension [122]. Recently, it has been shown that the *ACE* gene and smoking were related to plasma ACE levels and could potentiate the risk of atherosclerosis [113]. In fact, smoking and the *D* allele increase the degradation of NO and superoxide anion formation, which could cause endothelial dysfunction leading to the occurrence of CAD [122]. Amara et al., suggested that the *D* allele could be a predictive tool to determine who may be at risk of developing CAD. Indeed, the *ACE D*/*D* genotype increased the odds for CAD by more than eight-fold compared to the control (without the *D*/*D* genotype). In addition, it seems that the *D*/*D* genotype acts synergistically with hypertension, essentially as well as smoking, diabetes and family history of CAD and dyslipidemia, hence favoring the risk of coronary disease [122]. Vladeanu et al., have performed *ACE* genotyping among 154 patients with acute coronary syndrome, divided into four groups according to the severity of their vessel lesions, and then correlated genotypes obtained with the severity of the vessel-disease and the exposure to classic risk factors. They found an increase in the severity of acute CAD and severe coronary stenosis along with the ACE *D*/*D* genotype, while the *I*/*I* genotype was suggested to have a protective effect on the coronary arteries. Moreover, patients with the most severe vessel-disease and having the *D*/*D* genotype showed dyslipidemia and diabetes, with 50% of them being heavy smokers and obese [124].

The A1166C polymorphism of the *AT1R* gene may lead to the occurrence of hypertension, CAD and other CVD by increasing vasoconstriction and responsiveness to Ang II, as well as by cardiac and vascular hypertrophy [125]. Niemiec et al., concluded that the *AT1R* 1166C allele, along with some traditional risk factors of CAD (cigarette smoking and hypercholesterolemia), act synergistically to increase the risk [125]. Szolnoki et al., have shown that the *AT1R* A1166C polymorphism might lead to ischemic stroke indirectly via a deleterious effect on the cardiorespiratory function. In fact, the *AT1R* 1166C allele was associated with an increased risk of ischemic stroke (OR 22.3, 95% CI 5.8–110.2, *p* < 0.001) in hypertensive smokers. However, alone, this polymorphism did not pose a stroke risk [74]. Chandra et al., have studied, in a North Indian population, the association between *AT1R* A1166C gene polymorphism, its expression at transcript and protein levels and essential hypertension. They showed that the *A*/*A* genotype was the most dominant in the control group compared to *C*/*C* and *A*/*C* in the same group, whereas the *A*/*C* genotype was the most frequent among patients. They have also reported a 15.6-fold up-regulation of *AT1R* gene expression and a significant expression increase at the protein level, up to 1.9 times in patients with essential hypertension, compared to controls. The most important finding was that *AT1R* gene expression was higher in *C*/*C* genotype patients compared to *A*/*A* and *A*/*C*, which was not the case in the control group [38].

The association of *ACE I*/*D* and *AT1R* A1166C polymorphisms favors and enhances the Ang II/AT1R axis, thus contributing to circulatory problems, due to the mutualistic effect between the two polymorphisms [74,95]. Sekuri et al., studied the association of *AGT*, *ACE* and *AT1R* gene polymorphisms with pre-mature coronary heart disease (CHD) in a Turkish population. They showed that patients with *ACE D*/*D* and *AGT M*/*M* genotypes had a higher frequency of premature CHD, whereas *AT1R A*/*A* genotype subjects had the lowest risk. As for the *AT1R* 1166C allele, it was related to a higher risk of CHD [120]. Therefore, that study highlighted the synergistic effects of *ACE D*/*D* and *AGT M*/*M* polymorphisms in the contribution to premature CHD [120]. Borzyszkowska et al., examined whether the three RAS gene polymorphisms: *ACE* c.2306-117_404 *I*/*D*, *AT1R* c.1080*86A > C and *CYP11B2* (encoding for aldosterone synthase) c.344C > T are associated with the extension of coronary lesions in a group of 647 patients. An association between the *ACE I*/*D* polymorphism and the extension of atherosclerosis was shown among men with high total cholesterol levels (>200 mg/dl), low HDL cholesterol levels (≤40 mg/dl) and high LDL cholesterol levels (>130 mg/dl). However, no association between the other two polymorphisms has been found [127]. Szolnoki et al., examined the synergistic effect of *AT1R* A1166C with the ACE *D*/*D* polymorphism on the evolution of ischemic stroke. It has been seen that the co-occurrence of at least one *AT1R* 1166C allele with the homozygous *ACE D*/*D* was more frequent in ischemic stroke patients compared to normal ones. Therefore, these polymorphisms altered the RAS through a mutual interplay and were associated with the development of small-vessel ischemic stroke [95].

Moreover, *ACE* A11860G (rs4343), *ACE I*/*D* (rs4340), *AGT* T174M (rs4762), *AT1R* A1166C (rs5186) and *AGT* M235T (rs699) gene polymorphisms were analyzed by Freitas et al., in 510 controls and 298 CAD patients from Portugal. Results showed that the simultaneous presence of *ACE I*/*D* and ACE11860 A alleles increased the risk of developing CAD. In addition, the *D*/*D* genotype of *ACE* along with some classical risk factors (hypertension, obesity, diabetes and dyslipidemia) further increase the frequency of developing CAD. In addition, AT1R1166 interacts positively with obesity, smoking and hypertension, and the AGT235 *T*/*T* increases the CAD risk in the presence of hypertension and dyslipidemia [123]. Therefore, a SNP could have a significant association with the risk of a multifactorial disease [125].

### 3.5. COVID-19 and Diabetes

Diabetes is considered an essential underlying comorbidity in COVID-19 patients and was shown to induce mortality in these patients [128]. The initial case series of COVID-19 hospitalized individuals in many countries revealed over-representation of people with diabetes [129]. In fact, diabetes hyperglycemia may dysfunction and weaken the immune system, which then fails to encounter the invasive pathogens [69,128]. Akbariqomi et al., conducted a retrospective study on 595 COVID-19 hospitalized patients, out of which 148 diabetic patients showed high comorbidity (dry cough, fever and dyspnea) and mortality rates, especially in obese and hypertensive, compared to non-diabetic ones [128]. McGurnaghan et al., have shown that the risk of critical care unit-treated COVID-19 is increased by 1.4 and 2.4 times in type 2 and type 1 diabetes, respectively. Therefore, patients with recent hypoglycemia and diabetic ketoacidosis history have an increased risk of severe disease [54]. Similarly, the study conducted by Verma et al., showed that patients with diabetes are at increased risk (2.8-fold) of having severity or mortality from COVID-19. According to COVID-19 severity, they have classified those patients into two groups: 8.7% mild and 28.3% severe cases [69].

Interestingly, in rs4343 and rs4341 polymorphisms of the *ACE* gene, the presence of the G allele worsens COVID-19 outcome in diabetic patients, since patients admitted to the ICU had the GG genotype. However, these two polymorphisms are also related to the risk of developing severe COVID-19 complications requiring ICU admission in patients with hypertension and dyslipidemia. Thus, the G-containing genotypes confer a risk factor of having much more severe forms of COVID-19, independently of the gender, and are associated with higher death risks. Thus, the genotyping of these polymorphisms could indicate appropriate clinical management at hospital admission [36].

The effect of *I*/*D* polymorphism in the 16th intron of the *ACE* gene on metabolism has been investigated. In fact, *ACE I*/*D* was shown to be associated with metabolic syndrome [37]. Abdollahi et al., demonstrated that the *C* allele and, especially, *C*/*C* genotype were associated with lower insulin levels and fasting glucose levels at 30 min and 120 min, being particularly significant in men [37].

### 3.6. COVID-19 and Obesity

Obesity is due to an excess of food intake or altered energy expenditure; it is a condition that is implicated in many other diseases such as atherosclerosis, hepatic steatosis and metabolic syndrome, with increased insulin resistance that could evolve into type 2 diabetes [130]. There is emerging evidence that SARS-CoV-2 illness and disease severity are associated with overweight and obesity. A significant association between obesity and disease severity and mortality was already reported for other respiratory virus pandemics, including that of H1N1 influenza in 2009 [131,132].

Obese patients suffer more frequently from cardiovascular dysfunction and hypertension, and many of them have type 2 diabetes. In overweight and obese individuals, macronutrient excess in the adipose tissues stimulates adipocytes to release TNF-α, IL-6 and other pro-inflammatory mediators [133,134]. Obese patients also have a higher level of leptin, which is a pro-inflammatory adipokine, and a lower concentration of adiponectin, which is an anti-inflammatory adipokine, thus predisposing them to a pro-inflammatory state and oxidative stress [135]. IL-6 and TNF-α serum levels remained independent and significant predictors of disease severity and deaths of COVID-19 patients [136,137].

After cell invasion, the SARS virus induces a systemic downregulation of ACE2 to prevent another viral infection of the cell [50]. As the cardiovascular and anti-inflammatory protective factor of the ACE2/Ang-(1–7)/MasR axis is lacking, the balance shifts to the pro-inflammatory side [138], also explaining the cytokine storm that follows, which is a synergistic effect of the physiological condition of adipose tissue and pathological virus-induced reduction in ACE2 [139]. The higher expression of ACE2 in the adipose tissue favors SARS-CoV-2 penetration. Symptomatic obese patients shed the virus for a 42% longer time than non-obese subjects [140], and require longer hospitalization and more intensive care [141].

Given the extremely high rates of obesity around the globe, it is expected that a high percentage of the population infected with coronavirus will also have a BMI >25. A retrospective analysis of BMI in USA SARS-CoV-2 patients revealed that subjects aged <60 years with a BMI between 30 and 34 were 2 and 1.8 times more likely to be admitted to acute and critical care, respectively, compared to individuals with a BMI <30; while patients with a BMI >35 and aged <60 years were 2.2 and 3.6 times more likely to be admitted to acute and critical care compared to same-aged patients with BMI <30 [142]. Furthermore, a retrospective case-control study of young Chinese patients with COVID-19 showed that obesity was the most important critical factor contributing to their death [143]. In Germany, patients with ARDS were more commonly overweight or obese (83%) versus those with a normal BMI (42%) [144]. Therefore, obesity may be associated with COVID-19 morbidity and mortality independently of older age and the presence of comorbidities [143].

When interpreting the potential role of obesity as a risk factor for morbidity and mortality from COVID-19, the role of cardiovascular disease, hypertension and diabetes cannot be ignored, given that obesity is closely related to these conditions, all of them associated, as described above, with severe cases of COVID-19.

### 3.7. COVID-19 and Vitamin D Deficiency

Vitamin D, a steroid and versatile hormone, plays crucial roles in phosphorus–calcium metabolism and in the immune system of both humans and animals. Numerous studies have revealed a wide range of pharmacological and physiological functions of Vitamin D, among them anti-inflammatory, antioxidant and antiviral effects [145].

A recent genomics-guided tracing of SARS-CoV-2 targets in human cells identified vitamin D as a molecule manifesting potential infection mitigation patterns through their effects on gene expression. This is particularly achieved through the direct binding of vitamin D to the vitamin D response elements (VDRE) located in the promoter regions of several genes, the expression of which is either activated or repressed. Such a transcriptional regulation scheme involving vitamin D theoretically results in prevention of COVID-19 adverse outcomes by regulating many determinant aspects, including RAS, innate and adaptive cellular immunity and physical barriers [146].

COVID-19 infection is characterized by an increase in pro-inflammatory cytokine production which triggers the “cytokine storm” in severe cases, leading to lung and systemic inflammation. Elevated serum levels of IL-6 and other pro-inflammatory cytokines, such as TNF-α and IFN-γ, are hallmarks of systemic inflammation of COVID-19, contributing to disease severity and adverse clinical outcomes [52]. Therefore, modulation of the inflammatory response has been suggested as a potential therapeutic strategy [147]. Vitamin D may modulate inflammatory response through its effects on innate and adaptive immunity; it reduces the cytokine storm by reducing the production of pro-inflammatory cytokines and increasing the expression of anti-inflammatory cytokines by macrophages [148]. Calcitriol, the active vitamin D metabolite, targets macrophages, activates B and T cells, through vitamin D receptors, and induces immunoglobulin and cytokine production [51].

Vitamin D status appears to be strongly associated with COVID-19’s clinical severity. It has been demonstrated that Vitamin D deficiency is quite common among COVID-19 patients. A recent clinical study showed that COVID-19 patients supplemented with Vitamin D had less severe symptoms of the disease [149]. A low serum concentration of 25-hydroxyvitamin D (25(OH)D) was associated with impaired respiratory health and increased susceptibility to acute respiratory infections [148]. Among patients with COVID-19, 67 to 85% experience ARDS, which is one of the leading causes of mortality [150]. Previous studies reported that vitamin D deficiency was ubiquitous in patients with ARDS, while serum 1,25(OH)2D was higher in survivors compared to non-survivors [151]. A meta-analysis of 25 randomized controlled clinical trials showed that vitamin D supplementation prevented acute respiratory infections by 12%, mostly in those with low 25(OH)D levels at baseline [148].

A quasi-experimental study on 66 residents with COVID-19 from a French nursing home revealed that 82.5% of participants in the intervention group who received bolus vitamin D3 supplementation (during COVID-19 or in the preceding month) have survived, compared to only 44.4% in the comparator group corresponding to all other participants. The full-adjusted hazard ratio for mortality, according to vitamin D3 supplementation, was HR = 0.11 [95 %CI: 0.03; 0.48], *p* = 0.003. Kaplan–Meier distributions showed that the intervention group had a longer survival time than the comparator one [152].

Vitamin D also promotes the degradation of SARS-CoV-2 through autophagy mechanisms via an acidification process of endolysosomes [153]. COVID-19 suppresses the production of ACE2 receptors, and research suggests that vitamin D stimulates ACE2, which can bind to SARS-CoV-2 and prevent it from attaching to ACE2 receptors [154,155]. For preventive and treatment purposes, supplementation with Vitamin D among COVID-19 patients will halt the progression of the disease and subsequent mortality.

## 4. Conclusions

The SARS-CoV-2 outbreak has threatened several sectors in the world, including the economic and public health structure, and has furthermore caused hundreds of thousands of confirmed deaths, observed especially in elderly and immunocompromised people. Many studies are currently ongoing to understand the molecular mechanisms of COVID-19 cellular invasion, and thus the cascade activation and/or suppressing action detected among host cells. The normal signaling pathway of the RAS is clearly disturbed during viral infection due to the downregulation of ACE2 and the activation of Ang II/AT1R axis, which favors a pro-inflammatory state leading to cytokine storm, immunothrombosis, ARDS and multiple organ damage in COVID-19 patients. In addition, the manifestations and poor outcome of infection seem to be controlled by several risk factors, the presence of comorbidities and the genetic background of each individual. As a treatment, some brands worked hard to design a new vaccine that would protect the population and decrease the disease’s severity after taking two or more doses. More than 5.85 billion vaccines have been administrated now across 184 countries, according to some data. However, knowing that COVID-19 regularly enters into a mutation cycle, more efforts should be made on more efficient treatments to defeat SARS-CoV-2 definitely. Therefore, targeting the RAS could be an important therapeutic challenge in order to try to restore its equilibrium. In other words, several possibilities could be pointed out for future treatment of COVID-19, including (1) the use of stimulators or agonists of the protective pathway components to favor their function, such as an ACE2 protein-based vaccine or a recombinant ACE2, AT2R or MasR stimulator, or (2) the use of inhibitors targeting the Ang II/AT1R axis, such as AT1R blockers or Ang II antagonists. Therefore, these promising RAS therapeutic treatments aiming at reducing or even preventing COVID-19 diseases should be well studied in order to be supported by valuable conclusive data for their definitive usage.

## Figures and Tables

**Table 1:** Human gene mutations and polymorphisms of the Renin–Angiotensin System associated with COVID-19.

RAS Component Gene	Chromosomal Location	Associated Disease/Phenotype	Mutations, Polymorphisms and rs Number	Allele/Genotype Frequencies in Populations and Ethnicities	References
*ACE1*	17q23.3	CVD Kidney disease Autoimmune diseases Hypertension Hypercoagulability ARDS Type 2 diabetes Risk of obesity	Insertion/Deletion (I/D) of a 287-bp *Alu* repeat in intron 16 (rs1799752)	**Lebanese** *I*: 0.27, *D*: 0.73	[77]
				**Indians** *I*: 0.55, *D*: 0.45 **Whites** *I*: 0.5, *D*: 0.5 **African Americans** *I*: 0.41, *D*: 0.59	[76]
				**British** *I*: 0.31, *D*: 0.69 (ARDS) *I*: 0.49, *D*: 0.51 (healthy population)	[73]
				**Chinese** *I*: 0.705, *D*: 0.295	[75]
				**Italians** *I*: 0.342, *D*: 0.658	[56]
				**Italians** *I*: 0.27, *D*: 0.73	[35]
				**Germans** *I*/*I*: 0.27, *I*/*D*: 0.43, *D*/*D*: 30	[48]
				**Indians** *I*: 0.575, *D*: 0.425	[78]
*ACE2*	Xp22.2	Cardiovascular risk, Retinopathy in type-2 Diabetes Mellitus, Hypertension and Hypertensive left ventricular hypertrophy	c.*1860-449C > T SNP (rs2074192)	**Italians** *C*: 0.56, *T*: 0.44	[35]
			c.*264+788T > C (rs2106809)	**Italians** *A*: 0.77, *G*: 0.33	
			c.2115-268A > T SNP (rs233574)	**Africans** *C*: 0.92, *T*: 0.08 **Europeans** *C*: 0.67, *T*: 0.33 **East Asians** *C*: 0.996, *T*: 0.004 **South Asians** *C*: 0.814, *T*: 0.814 **Americans** *C*: 0.767, *T*: 0.233	[79]
			c.1402A > G p.Ile468Val SNP (rs191860450)	**East Asians** with an allele frequency (AF) = 0.011	[16]
			c.1022A > G p.Lys341Arg SNP (rs138390800)	**Africans** AF = 4 × 10^−3^	
			c.2191C > T p.Leu731Phe SNP (rs147311723)	**Africans** AF = 0.014	
			c.631G > A p.Gly211Arg SNP (rs148771870)	**Europeans** AF = 2 × 10^−3^ **South Asians** AF = 1.9 × 10^−3^	
			c.2089A > G p.Arg697Gly SNP (rs751603885)	**South Asians** AF = 2.4 × 10^−3^	
			c.2074T > C p.Ser692Pro SNP (rs14903946)	**Africans** AF = 6 × 10^−3^	
			c.55T > C p.Ser19Pro SNP (rs73635825)	**Africans** AF = 3 × 10^−3^	
*AGT*	1q42.2 1q42–43	Hypertension Heart failure Myocardial infraction	c.704T > C p.Met235Thr (aka Met268Thr) SNP (rs699)	**Tunisians** *M*/*M*: 0.291, *M*/*T*: 0.291 *T*/*T*:0.419	[80]
				**Vietnamese** *T*: 0.92, *M*: 0.08	[81]
				**Iranians** *T*: 0.39, *M*: 0.61	[82]
				**Indians** *M*: 0.52, *D*: 0.48	[78]
				**New Zealanders** *T*/*T*: 0.19, *T*/*M*: 0.47, *M*/*M*: 0.34	[83]
			c.521C > T p.Thr174Met SNP (rs4762)	**New Zealanders** *T*/*T*: 0.7, *T*/*M*: 0.2, *M*/*M*: 0.1	[83]
*AT1R*	3q21–q25	Systolic blood pressure Left ventricular hypertrophy Hypertension Aortic stiffness Myocardial infarction Carotid intimal-medial thickening, CAD and stroke, Overweight, Diabetes	c.1166A > C in the 3′ UTR SNP (rs5186)	**Egyptians** *C*:0.24, *A*:0.76 (control group) *C*:0.34, *G*:0.66 (premature CAD patients)	[84]
				**Jordanians** Higher frequency of *A* allele	[85]
				**Iranians** Higher frequency of *A* allele	[86]
*AT2R*	Xq23–26	Metabolic syndrome	−1332A > G SNP (rs1403543)	**Egyptians** *A*:0.55, *G*:0.45 (control group) *A*:0.41, *G*:0.50 (premature CAD patients)	[84]

## Data Availability

Not applicable.
